# Elucidating the Fitness of a Dead-End Trap Crop Strategy against the Tomato Fruitworm, *Helicoverpa armigera*

**DOI:** 10.3390/insects12060506

**Published:** 2021-05-31

**Authors:** Purushottam Gyawali, Shaw-Yhi Hwang, Paola Sotelo-Cardona, Ramasamy Srinivasan

**Affiliations:** 1 Department of Entomology, College of Agriculture and Natural Resources, National Chung Hsing University, 145 Xingda Road, South District, Taichung City 402, Taiwan; gyawalipg93@gmail.com (P.G.); oleander@dragon.nchu.edu.tw (S.-Y.H.); 2 World Vegetable Center, 60 Yi-Min Liao, Shanhua, Tainan 74151, Taiwan; paola.sotelo@worldveg.org

**Keywords:** *Solanum lycopersicum*, tomato fruitworm, cotton bollworm, oviposition, *Solanum viarum*, dead-end trap crop

## Abstract

**Simple Summary:**

The tomato fruitworm, *Helicoverpa armigera* Hübner (Lepidoptera: Noctuidae)*,* is a destructive polyphagous insect pest of tomatoes and its control largely depends on chemical pesticides. However, indiscriminate use of chemical pesticides has resulted in the development of resistance and posed serious environmental problems. Alternatively, in search of environmentally friendly pest management techniques, the use of trap crops has recently gained more attention. In this study, we compared different accessions of *S. viarum* to investigate which of them have the highest potential as a dead-end trap crop for *H. armigera*. Results from the laboratory experiment showed a significant variation among the *S. viarum* accessions in terms of *H. armigera* oviposition, and exhibited a higher level of resistance against *H. armigera* larvae compared to the tomato plants. Under the semi-field condition, trap cropping of *S. viarum* significantly reduces the total egg-laying of *H. armigera* on tomato. This study provides important information about the abilities of *S. viarum* plants to influence the life parameter of *H. armigera* and highlighted the possibilities to use *S. viarum* as a dead-end trap crop for the management of *H. armigera*. However, the efficacy of *S. viarum* needs to be tested under large, open-field conditions.

**Abstract:**

*Solanum viarum* has been proposed as a potential dead-end trap crop for the management of *Helicoverpa armigera* because of its unsuitability for larval growth and survival despite being overwhelmingly preferred for oviposition. This study delved into the different *S. viarum* accessions for ovipositional preference and non-suitability for larval growth and survival of *H. armigera*. Besides, foliage trichomes, acylsugars, and phenolic content of *S. viarum* plants were assessed and compared with tomato. Since there is no significant variation in the ovipositional preference and larval performance of *H. armigera*, our result revealed that all those evaluated accessions of *S. viarum* have the potential to be used as a dead-end trap crop for the management of *H. armigera*. However, significant variation among the *S. viarum* accessions in terms of *H. armigera* oviposition was also evident in a no-choice experiment. Because of high-density glandular trichomes, acylsugars, and phenolic content, *S. viarum* significantly impaired *H. armigera* larval growth and survival compared to the tomato. Hence, our study elucidated that the *S. viarum* plant fits with the criteria for dead-end trap crop, and has the potential as a dead-end trap crop for the *H. armigera*, which needs to be tested under large, open-field conditions.

## 1. Introduction

Tomato (*Solanum lycopersicum* L.) is one of the most extensively consumed and widely grown vegetables [1], produced on 5 million hectares with a production of more than 180 million tons globally [2]. As a tropical and subtropical crop, the commercial cultivation of tomato is constrained by several insect pests, which not only cause yield losses but also make fruits unfit for human consumption by damaging fruit quality [3]. 

The tomato fruitworm, also known as the cotton bollworm, *Helicoverpa armigera* Hübner (Lepidoptera: Noctuidae) is a destructive, highly-mobile, and polyphagous insect pest feeding on tomato, as well as 200 other plant species [4], including high-value crops such as tobacco, chickpea, maize, pigeon pea, and cotton [5]. Due to the destructive nature and worldwide distribution, its control largely depends on chemical pesticides [6], which at the same time may result in the development of insecticide resistance [7,8]. With the ability of an insect to develop pesticide resistance combining with the negative impacts on human and environmental health [5], the development of a sustainable pest management strategy becomes more challenging [9]. Therefore, this challenge has stimulated researchers’ interest in the development of better management alternatives by exploiting the reproductive and feeding behavior of the pest insects [3,10]. Due to insect pests having specific preferences for certain host plant species/cultivars and growth stages in response to sensory cues (i.e., olfactory, tactile, and visual), these specific preferences can be exploited in terms of management by using trap crops [11]. Trap cropping is a safe and sustainable pest management technique within the integrated pest management (IPM) strategy. It involves habitat manipulation of the pest species in the agricultural crop fields [12], on which specific plant species (i.e., trap crop) are grown in the proximity of the main crop in order to attract pest species and where insects will fail to survive or reproduce [13]. 

*Solanum viarum* Dunal (Solanaceae), commonly known as a tropical soda apple, is an economically and medicinally important plant in some parts of Africa and Asia, and hence is also known as Medicinal Solanum [14]. However, it is an invasive perennial weed [15,16], native to South America, and distributed to other parts of the world including Africa, North America, and Asian continents [17,18]. In South Asia, *S. viarum* is used as a medicinal plant due to a rich source of an alkaloid (Solasodine), from which steroid hormones can be synthesized for the treatment of several diseases [19].

For the first time in 1998, a heavy infestation of *H. armigera* on *S. viarum* was noticed during the screening of eggplant [*Solanum melongena* (Solanaceae)] and its wild relatives for the resistance to *Amrasca devastans* Distant (Hemiptera: Cicadellidae) at World Vegetable Center (WorldVeg), Tainan, Taiwan [20,21]. Several subsequent studies also confirmed that *H. armigera* strongly preferred *S. viarum* over a known host, tomato [5,17,22,23,24,25,26]. Given the choice between *S. viarum* and tomato, *H. armigera* female moths overwhelmingly preferred to oviposit on *S. viarum* plants due to the presence of plant volatiles containing several alkanes [25]. However, the larval development and survival was highly affected due to the presence of toxic allelochemicals such as glycoalkaloids in *S. viarum* leaves [26], as well as the presence of high-density glandular trichomes [27], which exude compounds like methyl-ketones and sesquiterpene hydrocarbons [24,25]. It was also reported that the undisclosed compound in the plants impeded *H. armigera* larval survival, growth, and development [5]. The strong preference of *H. armigera* for oviposition on *S. viarum* paired with the unsuitability for survival and development of the larval stage suggested that *S. viarum* could be used as a dead end-trap crop for the management of this important pest [25]. 

The use of *S. viarum* plant that is highly attractive to *H. armigera*, and has a higher content of plant secondary metabolites and foliage glandular trichome confirms its effectiveness as a dead-end trap crop. Therefore, we compared different accessions of *S. viarum* to investigate which of them may have the highest potential as a dead-end trap crop for *H. armigera*. First, the ovipositional preference and larval performance of *H. armigera* were assessed under laboratory conditions to identify the most attractive and resistant accession(s) of *S. viarum*. Second, a semi-field experiment was conducted to assess the impact of the most attractive *S. viarum* accession(s) on the ovipositional dynamics of *H. armigera* in the presence of its natural host tomato. Additionally, foliage trichomes, acyl sugars, and phenolic content of *S. viarum* plants were assessed and compared with tomato. Consequently, the results from this study will provide important information for the development of a safe and sustainable pest management strategy against tomato fruitworm as an effective alternative to chemical control.

## 2. Materials and Methods

### 2.1. Plant Materials

All the experimental plants (*S. viarum* and tomato) were grown under controlled conditions inside the greenhouse (26 ± 1 °C temperature, 70 ± 10% R.H., and photoperiod: L14 and D10). A collection of twelve *Solanum viarum* accessions is on display at the Genetic Resources and Seed Unit (GRSU), WorldVeg, Tainan, Taiwan. At the time of the study, seeds of only eight *S. viarum* accessions were available. Hence, we included these eight accessions originating from Taiwan, India, Thailand, Laos, Malaysia, and Japan in the current study. The seeds of eight *S. viarum* accessions were obtained from the GRSU, WorldVeg, and tomato (Var.: Victoria) seeds were procured from the Known-You Seed, Kaohsiung, Taiwan and subject to the seed treatment. Seeds were sown in the 72-cell seedling trays filled with commercial growing media (King Root substrate, Dayi Agritech Co., Ltd., Pingtung, Taiwan) and germinated in the WorldVeg greenhouse. Four weeks after sowing, seedlings were transplanted to the plastic pots (11 cm high and 12 cm diameter). The transplanted plants were watered twice a day and uniformly fertilized with chemical fertilizer (Foliar Nitrophoska) (N:P:K:Mg = 20:19:19:0.5; WangMa Enterprise Co., Ltd., Kaohsiung City, Taiwan) every two weeks. Eight weeks (after transplanting) old plants with similar leaf size and area were used in the laboratory and net-house experiments.

### 2.2. Insect

The *H. armigera* colony was maintained on an artificial diet containing kidney bean powder (9.85%), yeast powder (3.91%), wheat germ (3.61%), agar (2.4%), and other vitamins, antibiotics, and distilled water [28]. The insects were reared in laboratory conditions at 26 ± 1 °C temperature, 70 ± 10% R.H., and photoperiod: L14 and D10 at WorldVeg Insectary. Hatched larvae were placed into polystyrene cups with the artificial diet until the early third instar. Then, they were reared on diet individually in multi-cavity plastic trays with covers until pupation. Sexed pupae were placed in acrylic cylinders (15 cm diameter and 30 cm long) until emergence of the adult. Emerged adults were used for the experiment and some remaining adults were used to maintain insect colony for further generation. Adult insects were nourished with pure honey daily.

### 2.3. Multiple-Choice Experiment

In this experiment, the ovipositional response of *H. armigera* was evaluated using eight different accessions of *S. viarum*. The multiple-choice experiments were conducted under laboratory-controlled conditions (26 ± 1 °C temperature, 70 ± 10% R.H.) and using the BugDorm-2400 insect rearing tent of W75 × D75 × H115 cm (BD2400; MegaView Science Co. Ltd., Taichung, Taiwan). Insect rearing tents were used in the experiments and each tent was considered as a replication. This experiment was replicated 10 times, following a completely randomized design (CRD). A single plant from each *S. viarum* accession was positioned equidistantly inside a tent and arranged in a circle around the center of the tent. Two days old adult *H. armigera* moths at the rate of three pairs per replication (*n* = 3 female/tent) were released from the center. Honey drops and water-soaked cotton were placed for the nourishment of insects at the point of release. The total oviposition per plant was recorded and compared after three days of insect release.

### 2.4. Two-Choice Experiment

This experiment was conducted to compare the oviposition preference of *H. armigera* on *S. viarum* vs. tomato. In a two-choice test, a single plant of tomato (var. Victoria) and a single *S. viarum* plant per accession were placed into the BugDorm-2120 insect rearing tent (W60 × D60 × H60 cm) and provided with two pairs (*n* = 2 females/tent) of *H. armigera* adult moths from the center of the cage. This experiment was replicated five times, following CRD experimental design and under laboratory conditions (26 ± 1 °C temperature, 70 ± 10% R.H.). Honey drops and water-soaked cotton pieces were provided for the nourishment of adult insects. The total oviposition on both plants was recorded and compared after three days of insect release. 

### 2.5. No-Choice Experiment

In a no-choice test, eight different accessions of *S. viarum* were evaluated for the *H. armigera* oviposition. In this experiment, two pairs (*n* = 2 females/tent) of mature *H. armigera* adult moths were released in a BugDorm-2400 insect rearing tent (W75 × D75 × H115 cm) with a plant from each accession of *S. viarum* in the laboratory condition. This experiment was replicated 5 times. Total oviposition per plant was recorded after three days of insect release. 

### 2.6. Effect of S. viarum Plant Volatiles on Oviposition

The effect of host plant volatiles on the oogenesis of *H. armigera* was assessed for eight accessions of *S. viarum* and tomato as a check. For that purpose, a tomato plant was kept in a BugDorm-2120 insect rearing tent (W60 × D60 × H60 cm) together with the plant treatment (i.e., *S. viarum* accession or tomato) physically covered by a black cloth. The black cloth was thin, small, and just enough to prevent female moths of *H. armigera* from laying eggs on the covered plant but it allowed the release of host plant volatiles. Two pairs (*n* = 2 females/tent) of *H. armigera* adult moths were released from the center and nourished with pure honey. Total oviposition per tomato plant provided with or without *S. viarum* plant volatiles was recorded after three days of insect release. This experiment was replicated five times.

### 2.7. Trichome Density and Types

To compare the morphology of the glandular trichome present on different accessions of *S*. *viarum* and tomato plants, three different plants from each of the eight accessions of *S. viarum* and tomato (check) were simultaneously assessed for trichome density. The density of trichomes was counted from the interior middle-section of both (abaxial and adaxial) surface (mm^2^) of the third top-leaf of both plant species by using Leica S8 APO stereomicroscope (Leica Microsystems, Wetzlar, Germany) at 50×. Moreover, the procedure was performed via scanning Electron Microscopy with the top open leaf using a field emission electron microscope (JEOL JSM-633OF, Tokyo, Japan). The leaf samples were fixed with liquid nitrogen and were examined at 100× and the identification and classification of glandular trichomes were carried out based on the trichome morphology described by Luckwill (1943) [29].

### 2.8. Quantification of Acylsugar Content

Standard PGO (Peroxidase/glucose oxidase) based acylsugar assay was performed following the methodology described by Mutchler Lab, Cornell University, USA [30]. In brief, the topmost, smallest leaf from three different plants of each *S. viarum* accession and three lateral leaflets of tomato from the third internode towards apex were taken and placed into 50 mL plastic vials (12.5 K, RCF, Perform Ri Labcon Made in the USA). Immediately after collection of the leaf, a sample in a racked vial was kept in a drying oven until completely dried at 29 °C. When samples were completely dry, 5 mL of methanol was added, the vial capped, and it was washed by inverting a couple of times and hand swirling for a few seconds. One ml of the solution to each washed sample was taken out into the stripped tubes, sealed, and kept at −20 °C freezers until assay was performed. Simultaneously, washed dried leaf samples of the racked vials were placed in the hood until completely dry and leaf dry weight was recorded. At the same time, the standard solution was made with different concentrations of sucrose.

One hundred µL from each sample was transferred to the 96 well assay microtiter plate with 100 µL of 6 M ammonium hydroxide. The plate was sealed and incubated overnight in the fume hood. The next day, the samples were unsealed and allowed to dry down in the fume hood until completely dry. After complete drying of the plate, 200 µL of PGO reagent with oxidase enzyme was added to each sample and incubated for 3 h at room temperature (25 ± 1 °C). Gentle rotation of an orbital shaker (OS701, KS, Taiwan) was provided to facilitate uniform color development. Then, the samples were analyzed using a spectrophotometer at 490 nm, and absorbance data was recorded and dried leaf weight data were taken. The absorbance data onto the standard solution was used to calculate the standard curve that resulted in the linear equation (y = aX − b) with an R^2^ value of more than 0.98 or more. Based on that regression equation, the total acylsugar content was quantified and recorded as a micromole of acylsugar per gram (µmol/g) of leaf dry weight.

### 2.9. Quantification of Total Phenolic Content (TPC)

The total foliar phenolic content (TPC) of *S. viarum* and tomato was measured by following the Folin-Denish method [31,32]. The top third and fourth leaf from a different plant of each *S. viarum* accession and lateral leaflet of tomato from the third internode towards apex was taken. All the collected leaf samples were dried in a drying oven at 45 °C for two days, and dry leaf weight was taken and ground the leaf sample using a coffee grinder. Precisely 0.1 g of each ground sample was weighed and kept in two glass tubes; 9.9 mL of 100% methanol was added and the mixture was shaken on an orbital shaker for 4 h at high speed. The combined extract was centrifuged at 6000 rpm for 10 min. Around 7 mL of centrifuged sample was taken and stored at −70 °C. Simultaneously, 1 mM Chlorogenic-acid standard was freshly prepared by dissolving 17.7 mg chlorogenic acid into 50 mL methanol and further diluted to provide a series of concentrations of zero, 0.2, 0.4, 0.6, and 0.8 mM and the calibration line was constructed. 200 µL of Chlorogenic-acid standard and extracted leaf solution were kept in a test tube separately and 3.2 mL of double-distilled water was added followed by 200 µL of 1 N Folin-Ciocalteu’s phenol reagent into each test tube and solution was vortexed for mixing well. After mixing, 400 µL of a sodium carbonate solution was further added into the mixture and again vortexed. The final mixtures were incubated in the dark place at ambient room temperature for at least 30 min and the absorbance was determined using a spectrophotometer at 760 nm wavelength. Absorbance data onto standard solution was used to calculate the standard curve that resulted in the linear equation (y = aX − b) with an R^2^ value of more than 0.9950. Based on that regression equation, the total phenolic content was quantified and recorded as a milligram of phenols per 100 g (mg/100 g) of leaflet dry weight.

### 2.10. Feeding Experiment for the Larval Survival and Growth Analysis

For this experiment, young larvae were reared to the second instar in plastic cups containing the artificial diet. They were then transferred to the multi-cavity insect rearing tray at a rate of 20 larvae per treatment. Larvae were fed with the fresh but detached leaves of *S. viarum* and tomato until pupation. The control group of larvae was allowed to grow on an artificial diet. Each treatment was replicated 3 times following the CRD. Larval mortality, larval duration, pupation, and pupal weight were recorded and compared.

### 2.11. Oviposition on Tomato and S. viarum Mixtures

Oviposition experiments on tomato and *S. viarum* mixtures were conducted in the semi-field condition using 32-mesh nylon net houses (2.5 × 2.5 × 2 m), using a completely randomized block design with five treatments of a systematic mixture of tomato and *S. viarum* plants (0, 4, 8, 16, and 32% of plants were *S. viarum*) (*n* = 25 plants/treatment). The treatments were randomly assigned to each of these net houses and this experiment was replicated four times. All the experimental plants (tomato and *S. viarum*) were moved to the net houses from the greenhouses 24 h before releasing insects for the experiment. The *S. viarum* plants were placed at equidistant among the tomato plants, using the same arrangement in each replication. Two-day-old mated adults of *H*. *armigera* at the rate of 12 pairs per treatment (net houses) were released from the center of the net house using plastic containers placed at 80 cm above the ground level. Pure honey and water-soaked cotton were provided at the releasing point for the nourishment of adult insects. Three days after releasing adults, the eggs laid per plant were counted.

### 2.12. Statistical Analysis

Prior to statistical analysis, percentage data were *arcsine* transformed, and count data were square-root transformed to ensure the normality of the data. The differences in *H. armigera* oviposition preference on a multiple-choice experiment, no- choice experiment, volatile experiment, and oviposition on tomato and *S. viarum* mixture were analyzed by analysis of variance (ANOVA) with the Proc GLM procedure of SAS version 9.1 (SAS Institute, Cary, NC, USA). The two-choice oviposition experiment of *H. armigera* oviposition on *S. viarum* accessions and tomato were analyzed by paired t-test using online software GraphPad Prism 8. Differences in evaluated plant morphological (trichomes) and chemical (acylsugars and phenolics) characteristics and life parameters of the insect were analyzed by analysis of variance (ANOVA) with the Proc ANOVA procedure of SAS version 9.1 (SAS Institute, Cary, NC, USA). Mean separation was completed using Tukey’s Honest Significant Differences (HSD) post-hoc test and significant treatment differences were indicated. Finally, the correlations between different plant and insect life parameters were analyzed by IBM SPSS Statistics for Windows, Version 22.0 (IBM Corp, Armonk, NY, USA).

## 3. Results

### 3.1. Multiple-Choice Experiment

**Figure 1:**
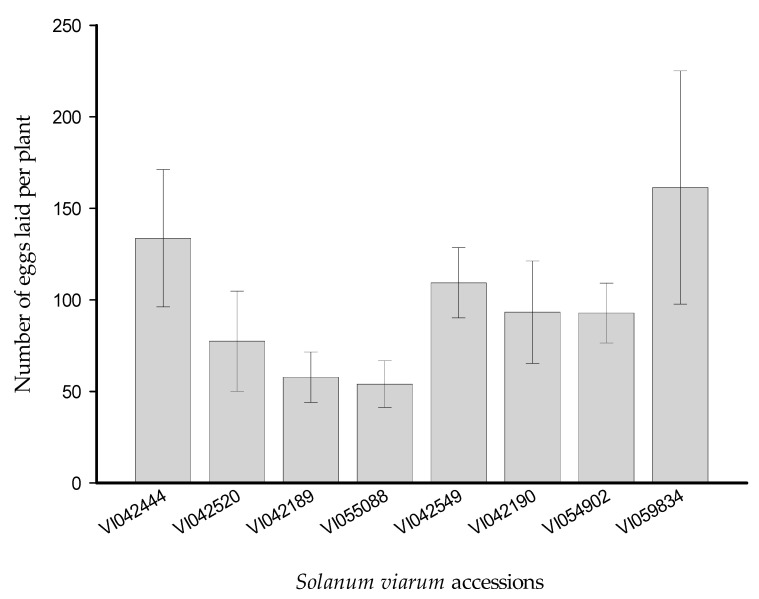
Number of eggs per plant (mean ± SEM) laid by *Helicoverpa armigera* female moths on different accessions of *Solanum viarum* plants (*n* = 10) under a multiple choice experiment.

The ovipositional response measured as the mean number of eggs laid per plant of *H. armigera* female moths did not differ significantly among the *S. viarum* accessions (*F* = 1.22, *df* = 7, *p* = 0.30) (Figure 1).

### 3.2. Two-Choice Experiment

In the two-choice assay, *H. armigera* gravid female moths showed significant preference to oviposit on three *S. viarum* accessions, VI042189, VI055088, and VI042190 compared to tomato (Table 1). The remaining *S. viarum* accessions did not statistically differ from tomato in terms of ovipositional preference.

### 3.3. No-Choice Experiment

**Figure 2:**
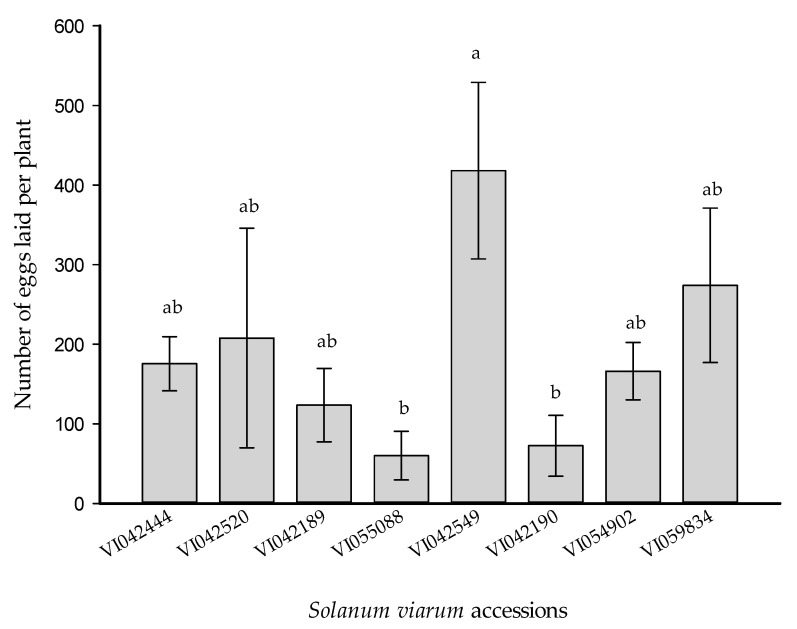
Number of eggs per plant (mean ± SEM) laid by *Helicoverpa armigera* female moths on *Solanum viarum* accessions (*n* = 5) under a no-choice experiment. Bars capped with the same letter(s) are not significantly different (*p* < 0.05).

Under the no-choice test, *H. armigera* females laid the highest mean number of eggs on the accession VI042549, which received 5.77 and 6.96 times more eggs compared to accessions VI042190 and VI055088, respectively (*F* = 2.58, *df* = 7, *p* = 0.036) (Figure 2).

### 3.4. Effect of S. viarum Plant Volatiles on Oviposition

**Figure 3:**
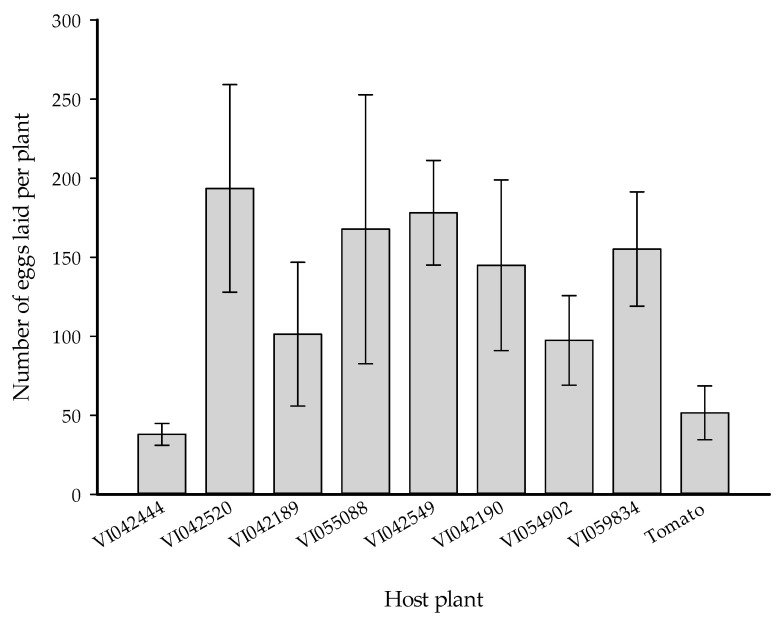
Number of eggs per plant (mean ± SEM) laid by *Helicoverpa armigera* female moths on a tomato plant in the absence or presence of volatiles from different *Solanum viarum* accessions (*n* = 5).

There is no significant difference in the total number of eggs laid by *H. armigera* on tomato plants provided with different *S. viarum* plant volatiles (*F* = 1.07, *df* = 8, *p* = 0.40) (Figure 3), and all tested *S. viarum* accessions showed an equal effect on *H. armigera* oviposition. 

### 3.5. Trichome Density and Types

We found significant differences among and between the evaluated *S. viarum* accessions and tomato in terms of glandular trichome density on both adaxial (*F* = 3.72, *df* = 8, *p* = 0.01) and abaxial (*F* = 17.33, *df* = 8, *p* < 0.0001) leaf surfaces (Table 2). A high density of glandular trichomes on the adaxial surface was observed in *S. viarum* accession VI042444, which is 2-fold higher than VI042549. In contrast, the *S. viarum* accessions VI042444, VI042190, and VI055088 recorded a significantly higher number of trichomes on the abaxial leaf surface than the other accessions. Thus, the accession VI042444 showed a significantly higher number of glandular trichomes on both leaf surfaces, whereas tomato contained the lowest density of glandular trichomes on both leaf surfaces in comparison to the *S. viarum* accessions. No significant difference (*F* = 1.75, *df* = 8, *p* = 0.155) was found in the adaxial non-glandular trichome density, but tomato showed a significantly higher number of abaxial non-glandular trichomes (*F* = 14.03, *df* = 8, *p* < 0.0001) than the *S. viarum* accessions.

**Figure 4:**
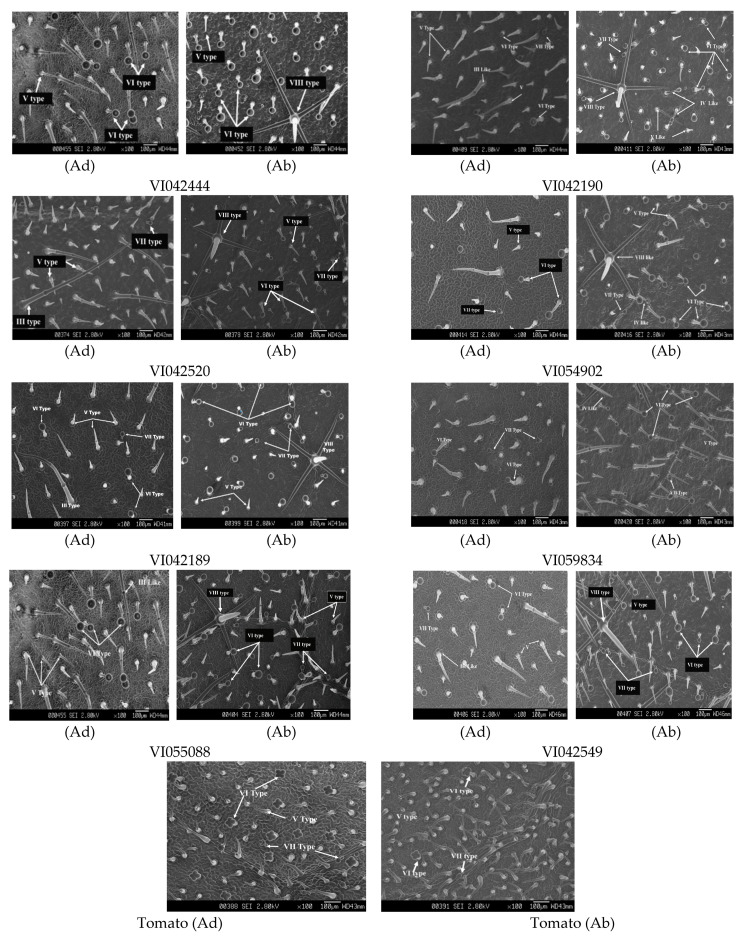
Scanning electron microscopic (SEM) pictures of trichomes on both leaf surfaces of *Solanum viarum* accessions and tomato (Ad—adaxial surface, Ab—abaxial surface).

Scanning electron micrographs (SEM images) of leaf trichomes on tomato and *S. viarum* are presented in Figure 4. The abaxial surface of *S. viarum* leaves is rich in glandular trichomes while high-density non-glandular trichomes were observed on the adaxial surface of the leaves. Almost all *S. viarum* accessions had a higher number of type VI trichomes (lobe-shaped, with both short and long stalk) and type VII glandular trichomes. The dendritic or stellar type branched (type VIII) trichome was observed only on the abaxial leaf surface of *S. viarum* accessions, but absent in tomato. The evaluated tomato plants had fewer glandular trichomes including type VI (four-celled) and type VII on the abaxial leaf surface, but a high number of non-glandular trichomes were observed in tomatoes.

### 3.6. Total Acylsugar Content

The total acylsugar content on the smallest leaves from the top of *S. viarum* plants was estimated and compared with the tomato leaflet taken from the third internode of the plant toward the top. The results revealed no significant difference in total foliage acylsugar content between *S. viarum* accessions and tomato (*F* = 1.96, *df* = 8, *p* = 0.113), (Table 3).

### 3.7. Total Phenolic Content (TPC)

*Solanum viarum* accessions had a 1.73 to 2.41-fold higher amount of total phenolic content than the tomato (*F* = 12.65, *df* = 8, *p* < 0.0001) (Table 3).

### 3.8. Larval Survival and Pupation on Different Accessions of S. viarum Leaves 

When early third instar larvae of *H. armigera* were fed with *S. viarum* accessions, tomato, and artificial diet as a control group, the results revealed significant differences in larval mortality (*F* = 4.61, *df* = 9, *p* = 0.002), larval development duration (*F* = 58.49, *df* = 9, *p* < 0.0001), and pupal weight (*F* = 105.42, *df* = 9, *p* < 0.0001) among the treatments (Table 4). However, there were no considerable differences in the life parameters of *H. armigera* among the *S. viarum* accessions. The larval duration of *H. armigera* on *S. viarum* was markedly longer than those on the tomato and artificial diet. Similarly, larval mortality on *S. viarum* accessions was significantly higher than those on tomato and artificial diet. The pupal weight of *H. armigera* fed on an artificial diet was twice as those fed with *S. viarum* leaves. Except for a few weak and abnormal adults, there was no adult emergence due to the deformed shape and size of pupae on *S. viarum,* while in an artificial diet, normal emergence of the adult was observed (data not shown). Thus, it was found that feeding on *S. viarum* leaves had a serious negative impact on larval survival and the growth of *H. armigera*.

### 3.9. Correlation Analysis Between H. armigera and Plant Parameters

The significant positive correlation of leaf glandular trichomes and total foliage phenolic compounds with the mortality and development of *H. armigera* larvae was evident in the current study, whereas the density of non-glandular leaf trichomes showed a significant inverse correlation. There is no evidence of a correlation or association between *H. armigera* oviposition and other plant parameters except for foliage trichomes (abaxial glandular and adaxial non-glandular). The weight of pupae formed on *S. viarum* was drastically reduced as a consequence of high-density glandular trichomes, but the opposite result was shown for the non-glandular trichomes. When we calculated the correlation between the evaluated plant parameters, the total foliage phenolic content was positively associated or correlated with the presence of high-density glandular trichomes while inversely correlated with the non-glandular trichomes density. Besides, we observed a fair correlation of larval mortality and oviposition of *H. armigera* with total foliage acylsugar content. Hence, the evaluated plant parameters have shown a significant correlation with the reproductive, developmental, and feeding behavior of *H. armigera* (Table 5).

### 3.10. Oviposition on Tomato and S. viarum Mixtures

**Figure 5:**
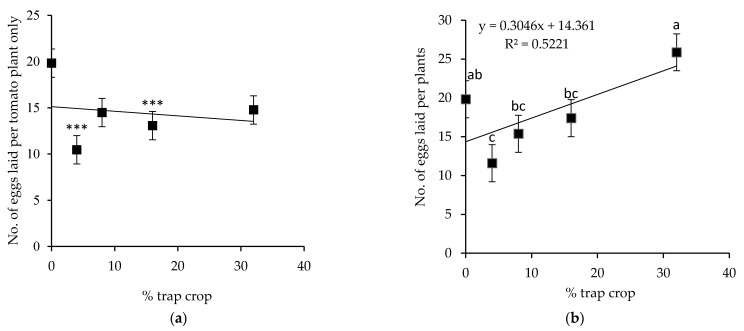
Number of eggs per plant (mean ± SEM) laid by *Helicoverpa armigera* female moths in tomato and *Solanum viarum* mixtures; (**a**) on tomato plant only, and (**b**) total no of eggs laid per plant on both tomato and *Solanum viarum* plants with an increasing percentage of trap crop. *p* < 0.0001 *** indicates highly significant differences compared to 0% trap cropping and bars capped with the same letter(s) are not significantly different (*p* < 0.05).

There was no statistically significant difference in the preference of *H. armigera* among the *S. viarum* accessions in the multiple-choice test. However, gravid females of *H. armigera* laid more eggs on accession VI042549 in the no-choice test (Figure 2). Therefore, this particular accession was selected for the mixture experiment. The net house (semi-field condition) experiment on *H. armigera* oviposition on tomato and *S. viarum* (trap crop) mixture recorded decreased numbers of egg per tomato plant in treatment with 4 percent and 16 percent trap crops (*F* = 5.99, *df* = 4, *p* ≤ 0.0001), whereas, for other treatments (8% and 32% trap crop), the difference was not significant compared to the tomato alone (Figure 5a). Also, the result of this experiment confirmed that the presence of *S. viarum* stimulated the total oviposition and oogenesis of *H. armigera* with the increased percentage of trap crop *S. viarum* (*F* = 8.66, *df* = 4, *p* ≤ 0.0001) (Figure 5b).

## 4. Discussion

In this study, we investigated ovipositional preference and larval performance of *H. armigera* on different accessions of *S. viarum* and tomato plants. The correlations of ovipositional preference and larval performance with the morpho-chemical characteristics (trichomes, phenolics, and acyl sugars) of the host plant were analyzed in order to determine the contributing factor that influences the pest performance on the specific host plant. Our result demonstrated that the female moth of *H. armigera* laid more eggs on the *S. viarum* over tomato plants, though on that host plant larvae performed poorest. Although *S. viarum* is a highly preferred host for the *H. armigera* oviposition, it exhibited a higher level of antibiosis against *H. armigera* caterpillars due to the presence of high-density glandular trichomes and a higher concentration of secondary plant metabolites (phenolic compounds and acylsugars) than the natural host tomato. Therefore, as we expected, our results were not consistent with “preference-performance hypothesis” that the female moth of herbivore insect will search or prefer a host that would most favorable for the growth and development of their offspring.

In general, as predicted by the preference-performance hypothesis, the reproductive fitness of a herbivore insect is very much dependent on the host selection behavior of the females and the nutritional availability of the host plant [33,34,35,36,37]. This hypothesis is also known as optimal oviposition theory and is considered the dominant paradigm in the studies of insect-plant interactions. However, in this study, the host preference of *H. armigera* for the oviposition was not in concordance with the survival ratio and larval development period. Therefore, the overall results of this study do not support the optimal oviposition theory. This may be due to the lower ability of a polyphagous insect to discriminate between the host plant with different quality as well as different ecological conditions and selection pressures. [33,38,39,40]. For instance, given the choice between resistant and susceptible maize cultivars, the female moth of fall armyworm (*Spodoptera frugiperda*) laid more eggs on the highly resistant cultivar [41]. In another study, the unsuitability of plant *Barbarea vulgaris* var. *arcuata* for larval survival of *Plutella xylostella* L. (Lepidoptera: Plutellidae) despite being overwhelmingly preferred for the oviposition was also reported and this particular phenomenon was termed as a dead-end trap cropping [10,11,13,42], which was purposed as an alternative pest management technique for the management of *Plutella xylostella* [11,13]. Accordingly, our study also confirmed that the *S. viarum* plant fits with the criteria of dead-end trap crop for *H. armigera* as defined by Shelton and Badenes–Perez [13].

### 4.1. S. viarum, an Oviposition Attractant for H. armigera

The host selection is a crucial decision of herbivore female insects, as the oviposition site fundamentally affects offspring’s survival, performance, and phenotype, as well as the reproductive success and potential survival of ovipositing females itself [36,41]. Therefore, the host selection for oviposition is a critical life-history trait for the polyphagous herbivorous insect especially for *H. armigera* because its neonate larvae are less mobile [33,36,37]. In terms of ovipositional preference, the absence of any significant variation in the multiple-choice and volatile experiment indicates an equal ovipositional preference of *H. armigera* on all the evaluated accessions of *S. viarum*. This output illustrated the equal impact of *S. viarum* accessions on *H. armigera* oviposition, and hence they all have the potential to be used as a trap crop. In contrast, the no-choice oviposition experiment showed significant variation among evaluated accessions of *S. viarum.* The gravid females of *H. armigera* laid more eggs on accession VI042549 compared to other evaluated accessions of *S. viarum*. It could be the most attractive or preferred accession than other evaluated accessions. The differences in ovipositional response could be due to the different experimental designs, experimental conditions, density, and the spatial distribution of host plant species, and the number of female moths released [42]. Additionally, the presence of flowers, plant height, plant nutrient supply, and chemical attractant of the host plants have a significant impact on *H. armigera* oviposition [43]. Similarly, temperature influences reproduction, development, and the survival of the insect population [44,45]. The age of plants also influences ovipositional preference and attractiveness of host plants to insect pests [46]. However, Srinivasan et al. [25] concluded that the age of *S. viarum* plant is not a major influencing factor to affect the oogenesis and oviposition of *H. armigera*. Therefore, seasonal impact (summer) and tropical climate of our experimental location combined with controlled (temperature and wind flow) laboratory conditions may not have provided the best condition for mating, host selection, and fecundity of *H. armigera* moths, which could explain the intra-accession variations among the replications and high deviation of the obtained data. 

Furthermore, in two-choice assays, *S. viarum* accessions VI042189, VI055088, and VI042190 showed a significantly strong attraction for *H. armigera* oviposition, which is supported by the previous findings of Srinivasan et al. [25] on which, *S. viarum* plants were also overwhelmingly preferred by *H. armigera* over its natural host tomato. In general, the female moth of *H. armigera* prefers to lay eggs singly or sometimes in bunches and clusters, especially on a vigorously growing top third portion of host plants [47]. But in this study, we observed that the female moth of *H. armigera* dumped their eggs everywhere (leaves, stem, stem base, plant growing media, pot, and wall of the insect rearing cage) singly, in clusters, and even in big overlapping clusters while provided with the *S. viarum* plants. This altered reproductive behavior with enhanced oogenesis and oviposition of *H. armigera* female is indicating the presence of attractive and/or stimulating plant volatiles in *S. viarum.* For instance, several low molecular weight alkanes and 13, 17, 21-trimethylheptatriacontane (C_40_H_82_) and octacosane (C_28_H_58_) was reported in *S. viarum* leaves [26]. Previously, Diongue et al. [17] also reported about 21 plant volatiles (mainly higher alkanes) identified from the leaf cuticular extracts of *S. viarum* with evidence of increased fecundity and oviposition of *H. armigera*. A similar result of phytochemical stimulation was also illustrated by Burguiere et al. [48], where terpenoids (α-phellandrene and ocimene), a compound produced by the host plant elicited the highest electro-antennographic responses in young and virgin *H. armigera* female moths. Recently, in their study on the effect of plant volatiles on *H. armigera* oviposition, Srinivasan et al. [25] added strong evidence for plant volatile from *S. viarum* stimulating and enhancing the fecundity of female *H. armigera* moths. Although our result on the effect of plant volatiles on oviposition did not show any significant difference, higher egg deposition by *H. armigera* on *S. viarum* hints the impact of host plant volatiles on oogenesis and ovipositional behavior of *H. armigera*.

The semi-field experiment on the impact of the most attractive *S. viarum* accession (VI042549) on oviposition dynamics of *H. armigera* under semi-field condition showed that 4% and 16% trap cropping with *S. viarum* were able to reduce the total egg-laying of *H. armigera* on tomato plants considerably than in the treatment only with tomato. However, there is a lack of linear decrease in the numbers of egg deposition per plant corresponding to the increasing percentage of *S. viarum* trap crop. This difference in oviposition could be a consequence of the relatively confined enclosed area of the net house compared to the field, the spatial arrangement of the experimental plants, and the number and location of released insects [42]. In an earlier study, Badenes–Perez et al. [42] mentioned that despite the overwhelming preference of insects for a specific host plant, the distribution of egg deposition could be influenced by the limited availability of preferred host plants. Also, the density of moths within an experimental unit may lead to unusual and unexpected ovipositional behavior in the experiments [49], and this could be the case in the current study. The experimental conditions combined with the higher plant height of tomato compared to *S. viarum* used in this experiment may explain the high deviation in the recorded number of eggs laid by *H. armigera*. On the other hand, the linear increase in the number of eggs per plant with an increasing percentage of trap crop (*S. viarum*) in the mixture experiment over treatment with the only tomato indicated the presence of oviposition stimulants in *S. viarum* plants. Similarly, an earlier study on *H. armigera* oviposition in tomato and *S. viarum* mixture conducted by Srinivasan et al. [25] also found a similar result. However, in their study, Badenes–Perez et al. [42] reported that the percentage of trap crops directly correlated with the increased number of *P. xylostella* egg deposition per plant. The current study also emphasized that the host selection and oviposition of *H. armigera* on *S. viarum* are likely to be affected by several factors in addition to specific host plant volatiles. Hence, additional studies are required to confirm a similar effect on the large, commercial tomato field. 

### 4.2. Unsuitability of S. viarum for the Larval Survival and Development of H. armigera

In addition to oviposition, an earlier study confirmed the presence of feeding stimulants and the overwhelming preference of larvae to feed on *S. viarum* leaves than the tomato fruits and leaves [24]. However, larval growth and survival of *H. armigera* on *S. viarum* leaf-feeding were highly impaired [5,25]. Additionally, our study showed that larvae exposed to *S. viarum* leaves suffered higher mortality, an extended larval stage, and mostly deformed and significantly underweight pupae. No adult emergence, except a few deformed or abnormal adults (distorted wings, which were attached to the outer skeleton of the pupae), was also found in *S. viarum* leaf feeding, while in artificial diet normal adult emergence was observed. An earlier study also confirmed that the mortality and larval developmental time of *H. armigera* were increased on an artificial diet fortified with *S. viarum* leaves, along with the reduced pupal weight and mostly deformed shape of pupae [5]. Increased mortality and longer developmental time was further recorded by Srinivasan et al. [25] when they fed larvae with fresh young and old *S. viarum* leaves. This negative impact on larval development and survival of *H. armigera* might be due to the presence of a higher amount of toxic allelochemicals (Glycoalkaloids: Solasodine) in *S. viarum* leaves [50,51]. Insect mortality in the *S. viarum* was due to the presence of high density VI type-glandular trichomes that exude a higher concentration of methyl ketones and sesquiterpene hydrocarbons, which is associated with the host plant resistance against insect pests [52] and is especially toxic to *H. armigera* [24].

### 4.3. Morpho-Chemical Composition Vs. H. armigera Performance on S. viarum and Its Natural Host Tomato

There are seven morphologically different types of glandular (VI, VII, and IV) and non-glandular trichomes (II, III, V, and stellar trichome VIII type) present in *S. viarum* [27] that exhibit huge diversity in both types (glandular and non-glandular) of trichomes. In this study, our result also revealed significant variation not only in types, but also in the density, as well as their morphological structure of trichome in *S. viarum*. Furthermore, *S. viarum* accession VI042444 exhibited a significantly high density of foliage glandular trichomes on both (adaxial and abaxial) surfaces of leaves among the evaluated *S. viarum* accessions. In an earlier study on morphology and abundance of the trichome, Georgievska [24] observed radical differences between *S. viarum* and tomato. Variation in the presence of glandular trichome is positively correlated with the larval mortality and duration of the *H. armigera* larval period. However, there is no evidence found on trichome secreted and synthesized sesquiterpene stimulating the *H. armigera* oviposition [53]. Similarly, in this study, except for a negative correlation in a no-choice experiment, there is no significant correlation between glandular trichomes and *H. armigera* oviposition.

Apart from the direct role of plant structural traits such as trichomes, the plant tends to produce an immense number of defensive plant secondary metabolites in response to stresses (abiotic and biotic), which act as a signal compound [54], and physical or chemical deterrents that play a crucial role in resistance against herbivore insects [55,56]. Among 119 literature cited phytochemicals as to have insecticidal activity, the role of terpenoids, alkaloids, and phenolic compounds have been mostly considered due to the presence of their insecticidal and antifeedant property [57]. The acylsugar and phenolic compounds are the major, as well as most important, plant secondary metabolites that were evaluated in this study by considering their significant contribution towards the antifeedant and defensive role against herbivore insect pests. In their study, Ali et al. [58] observed a significant reduction in pupation on resistant tomato accessions, which could be due to the glandular trichome secreted toxic substances. The broad-spectrum insect resistance in *Solanum pennellii* against whitefly (*Bemisia tabaci*), and *Solanum galapagense* against two-spotted spider mite (*Tetranychus urticae,* Koch) has already been reported by several authors based on the presence of glandular trichome (especially IV type) and its exudates, especially acylsugar [1,59,60]. Similarly, *Solanum pimpinellifolium* (accession VI030462) was also found to have high resistance to tomato fruitworm (*H. armigera*), spider mites (*T. urticae*), tomato leaf miner (*Tuta absoluta*), and whiteflies (*B. tabaci*) due to the presence of toxic chemical compounds secreted by glandular trichomes [1,58,61,62]. In this current study, all the accessions of *S. viarum* appeared to have high acylsugar content than the tomato. The quantified acylsugar in our study also exhibited a moderate correlation with larval mortality. Therefore, acylsugar might have contributed to increased larval mortality of *H. armigera* in *S. viarum* leaf feeding. Even though trichome secreted acylsugar ingestion may not be lethal or toxic for herbivore insects’ larvae, it may lead to chronic larval mortality due to the adverse effect on growth and development [63]. Therefore, in our study, the result of a substantial reduction in pupation on *S. viarum* leaf-feeding could be the impact of antibiosis contributed by toxic exudates of glandular trichomes. 

Phenolic compounds also represent one of the most prevalent and common defensive chemical compounds, which play a crucial role in host plant resistance against herbivore insects [64]. The current study reported underlying differences between *S. viarum* and tomato plants in terms of foliage phenolic content, and its significant positive correlation with insect mortality, and glandular trichome density that emphasized the role of trichome on total phenolic content and their final impact on larval mortality of *H. armigera*. Moreover, *S. viarum* accession VI054902 was found to have a higher concentration of foliage phenolic content among evaluated *S. viarum* accessions. Earlier findings of Jayanthi et al. [27] also reported that the trichomes of *S. viarum* contained a higher amount of phenols. Additionally, in *S. viarum* trichome type VIII contributed to the highest amount of phenolic content, followed by type VII and type VI [27]. Since the current study recorded that *S. viarum* plants contained the highest number of these trichome types, it is not surprising to record a higher total phenolic content in *S. viarum* compared to the tomato. 

### 4.4. S. viarum as a Dead-End Trap Crop and Implications for H. armigera Management

This study confirmed that the *S. viarum* has potential to be used as a dead-end trap crop for *H. armigera* because of its unsuitability for larval growth and survival despite being preferred for oviposition. Utilizing a dead-end trap (*S. viarum*) as a border plantation or next to the main crop could help to manage the *H. armigera* on tomato sustainably by concentrating or accumulating the target pest population into a desired manageable area. These accumulated or trapped insect pests (adult, eggs, and/or larvae) on the trap crop can easily be destroyed or managed naturally due to its broad-spectrum resistance. Trap cropping is considered as the cultural method of pest control in the IPM, but it also allows the integration of other pest management/control strategies to manage the insect pest population under economic injury/damage level as well [65]. Although the *S. viarum* trap cropping limits the area of the main crop tomato, still farmers will be able to maximize their economic profit by utilizing the medicinal and economic value of trap crop *S. viarum*, along with the reduced economic damage on the main crop by *H. armigera*. However, the efficacy of *S. viarum* and its use as a dead-end trap crop in commercial fields of tomato remains to be investigated. There are regions in the America where *S. viarum* is listed as a noxious weed, so its potential use as a trap crop will vary. Therefore, precautions should be taken when using it as a trap crop. Furthermore, as mentioned by the previous researchers, the change in crop cultivation practices, such as density and placement of trap crop, planting date, and fertilization in the field has the potential to increase the performance and effectiveness of trap crop [10,42,46,66]. This could be the same case for *S. viarum*, which needs to be investigated by future studies. Further studies including additional defensive traits such as alkaloids (Solasodine) are necessary to explain the result and conclusion obtained from this study.

## 5. Conclusions

In conclusion, the overall results of this study are relevant from both ecological and agricultural pest management perspectives. From an ecological perspective, our results provide insights into host selection, oviposition preference, and larval performances of herbivore insects in different host plants under different growing conditions. Therefore, our study indicated a lower ability of a polyphagous insect pest to differentiate or discriminate between the host plant with different qualities. Hence, the adult females of herbivore insects may tend to choose relatively unsuitable host plants for their offspring. In addition, this study provides valuable information for an alternative pest management strategy by showing that *S. viarum* can be used as a dead-end trap crop for *H. armigera* due to its unsuitability for larval growth and survival despite being preferred for oviposition. 

## Figures and Tables

**Table 1:** Number of eggs per plant (mean ± SEM) laid by *Helicoverpa armigera* female moths on tomato and *Solanum viarum* accessions (*n* = 5), under a two-choice experiment. Figures in parentheses are square root (X + 0.5) transformed values. **p <0.001; *p < 0.05; NS *p* > 0.05.

Plant Species	Number of Eggs Laid Per Plant (Mean ± SEM)	t-Value	*p*-Value	Significance
*S. viarum* VI042444	104.00 ± 66.18	0.4454	0.6791	ns
	(8.28 ± 2.99)			
Tomato	55.60 ± 19.67			
	(7.04 ± 1.28)			
*S. viarum* VI042520	182.80 ± 101.59	1.0232	0.3641	ns
	(11.16 ± 3.83)			
Tomato	50.50 ± 18.45			
	(6.75 ± 1.18)			
*S. viarum* VI042189	362.00 ± 102.89	4.8531	0.0083	**
	(18.07 ± 3.00)			
Tomato	6.60 ± 3.84			
	(2.23 ± 0.73)			
*S. viarum* VI055088	580.00 ± 243.56	3.5657	0.0235	*
	(21.19 ± 5.75)			
Tomato	29.80 ± 23.83			
	(4.23 ± 1.76)			
*S. viarum* VI042549	180.40 ± 107.13	1.9046	0.1296	ns
	(11.54 ± 3.45)			
Tomato	17.80 ± 11.16			
	(3.47 ± 1.25)			
*S. viarum* VI042190	330.60 ± 165.82	3.2449	0.0315	*
	(16.20 ± 4.14)			
Tomato	23.00 ± 7.36			
	(4.39 ± 1.02)			
*S. viarum* VI054902	97.80 ± 45.06	1.2157	0.2909	ns
	(8.56 ± 2.50)			
Tomato	55.80 ± 41.07			
	(6.08 ± 2.20)			
*S. viarum* VI059834	281.80 ± 62.48	2.0954	0.1042	ns
	(16.38 ± 1.87)			
Tomato	103.60 ± 41.63			
	(9.00 ± 2.40)			

**Table 2:** Trichome density (mean ± SEM) on tomato and *Solanum viarum* accessions. Means within a column followed by the same letter(s) are not significantly different (Tukey’s HSD test (*p* ≤ 0.05).

Plant	Glandular Trichome Density/mm^2^ Leaf		Non-Glandular Trichome Density/mm^2^ Leaf	
	Adaxial	Abaxial	Adaxial	Abaxial
*S. viarum* VI042444	3.88 ± 0.15 a	7.12 ± 0.83 a	4.90 ± 0.36	4.37 ± 0.08 b
*S. viarum* VI042520	2.75 ± 0.12 ab	5.67 ± 0.77 ab	5.05 ± 0.36	4.83 ± 0.16 b
*S. viarum* VI042189	2.75 ± 0.23 ab	5.67 ± 0.15 ab	4.56 ± 0.30	4.93 ± 0.62 b
*S. viarum* VI055088	3.05 ± 0.42 ab	6.23 ± 0.17 a	5.47 ± 0.85	4.14 ± 0.37 b
*S. viarum* VI042549	1.88 ± 0.26 b	3.93 ± 0.17 b	3.53 ± 0.19	3.57 ± 0.27 b
*S. viarum* VI042190	2.95 ± 0.36 ab	6.25 ± 0.30 a	5.02 ± 0.96	4.37 ± 0.33 b
*S. viarum* VI054902	2.63 ± 0.25 ab	5.77 ± 0.48 ab	5.81 ± 0.92	5.52 ± 0.45 b
*S. viarum* VI059834	2.87 ± 0.54 ab	5.52 ± 0.45 ab	4.47 ± 0.29	5.09 ± 0.33 b
Tomato (check)	1.88 ± 0.26 b	0.60 ± 0.13 c	6.38 ± 0.75	11.15 ± 0.49 a

**Table 3:** Total acyl sugar (µmol/g of dry weight) and phenolic content (mg/100 g of dry weight) in the foliage of *Solanum viarum* accessions (*n* = 3) and tomato (*n* = 3). Means within a column followed by the same letter(s) are not significantly different (Tukey’s HSD test (*p* ≤ 0.05).

Plant	Total Acyl Sugar (µmol/g of Dry Weight)	Total Phenolic Content (mg/100 g of Dry Weight)
*S. viarum* VI042444	2.25 ± 0.23	2432.70 ± 192.65 ab
*S. viarum* VI042520	2.08 ± 0.05	2030.60 ± 242.89 b
*S. viarum* VI042189	2.04 ± 0.21	2568.42 ± 126.61 ab
*S. viarum* VI055088	1.79 ± 0.06	2384.26 ± 107.31 ab
*S. viarum* VI042549	2.26 ± 0.19	2298.66 ± 76.13 ab
*S. viarum* VI042190	1.81 ± 0.23	2674.01 ± 146.47 ab
*S. viarum* VI054902	2.45 ± 0.40	2825.50 ± 203.46 a
*S. viarum* VI059834	2.49 ± 0.12	2675.56 ± 136.49 ab
Tomato	1.68 ± 0.11	1170.67 ± 76.76 c

**Table 4:** Larval mortality, duration of larval development, and pupal weight of *Helicoverpa armigera* on *Solanum viarum* accessions, tomato leaves, and artificial diet. Means within a column followed by the same letter(s) are not significantly different (Tukey’s HSD test (*p* ≤ 0.05).

Treatments	Initial No. of Larvae	Larval Mortality (%)	Larval Development (Days)	Pupal Weight (mg)
*S. viarum* VI042444	100	55.00 ± 6.64 a	27.10 ± 0.79 a	119.14 ± 1.98 c
*S. viarum* VI042520	100	53.33 ± 4.41 a	25.73 ± 0.47 a	102.36 ± 2.55 c
*S. viarum* VI042189	100	55.00 ± 6.64 a	27.18 ± 0.56 a	116.43 ± 6.16 c
*S. viarum* VI055088	100	51.67 ± 6.67 a	25.25 ± 0.43 a	114.75 ± 6.00 c
*S. viarum* VI042549	100	56.67 ± 1.67 a	25.1 ± 0.22 a	119.73 ± 6.06 c
*S. viarum* VI042190	100	55.00 ± 7.64 a	25.43 ± 0.26 a	119.18 ± 0.93 c
*S. viarum* VI054902	100	60.00 ± 5.77 a	26.75 ± 0.46 a	110.13 ± 1.52 c
*S. viarum* VI059834	100	51.67 ± 7.26 a	26.38 ± 0.69 a	112.87 ± 3.99 c
Tomato	100	33.33 ± 1.67 ab	20.71 ± 0.50 b	175.76 ± 2.77 b
Artificial diet	100	21.67 ± 1.67 b	14.60 ± 0.59 c	234.59 ± 3.50 a

**Table 5:** The Correlation between *Helicoverpa armigera* oviposition, larval survival and development, pupal weight, and plant parameters (trichome density, total acylsugar, and phenolic content). (*n* = 9), (Pearson correlation **p < 0.01; *p < 0.05).

Correlation Between *H. armigera* Oviposition and Evaluated Plant Parameters.						
	Glandular Trichome		Non-Glandular Trichomes		Total Acylsugar Content	Total Phenolic Content
	Adaxial	Abaxial	Adaxial	Abaxial		
No-choice Test	−0.583	−0.774 *	−0.771 *	−0.288	−0.583	−0.774 *
**Correlation between *H. armigera* Life Parameters and Evaluated Plant Parameters**						
Larval mortality (%)	0.534	0.850 **	−0.696 *	−0.907 **	0.528	0.882 **
Larval duration (days)	0.389	0.811 **	−0.676 *	−0.963 **	0.448	0.830 **
Pupal wt. (mg)	−0.440	−0.851 **	0.499	0.895 **	−0.558	−0.881 **
**Correlation between Trichomes and Evaluated Plant Secondary Metabolites**						
Total acylsugar content	0.174	0.372	−0.456	−0.418		
Total phenolic content	0.505	0.830 **	−0.437	−0.792 *		

## Data Availability

The data presented in this study are available on request from the corresponding author. The data are not publicly available for a certain period of time and later can be accessed from https://worldveg.tind.io/ (accessed on 28 May 2021).
